# Serum YKL-40 levels and disease characteristics in patients with rheumatoid arthritis

**DOI:** 10.22088/cjim.10.1.92

**Published:** 2019

**Authors:** Mohammad Reza Jafari-Nakhjavani, Amir Ghorbanihaghjo, Babak Bagherzadeh-Nobari, Aida Malek-Mahdavi, Nadereh Rashtchizadeh

**Affiliations:** 1 Connective Tissue Diseases Research Center, Tabriz University of Medical Sciences, Tabriz, Iran; 2 Biotechnology Research Center, Tabriz University of Medical Sciences, Tabriz, Iran

**Keywords:** Rheumatoid arthritis, YKL-40, Disease activity

## Abstract

**Background:**

The present study aimed to evaluate serum YKL-40 levels in patients with rheumatoid arthritis (RA) compared to healthy subjects and to search whether there is an association between YKL-40 levels and disease characteristics in RA.

**Methods:**

In this cross-sectional study, 60 RA patients based on the ACR/EULAR 2010 criteria and 30 age- and sex-matched healthy controls were included. In patients, clinical examination was performed and disease activity score 28 (DAS-28) measure of disease activity was assessed. Serum YKL-40 level was measured using ELISA kit.

**Results:**

The mean±SD age of patients and controls was 54.86±11.65 and 50.71±3.72 years, respectively). Serum YKL-40 level was significantly higher in RA patients (951.63±639.98 pg/mL) compared to healthy controls (444.92±150.37 pg/mL) (P<0.001). There was no significant difference in serum YKL-40 level according to the activity of disease (p>0.05). There were significant positive correlations between serum YKL-40 level with disease activity (r=0.347, P=0.007) and rheumatoid factor (r=0.396, P=0.002). There were no significant correlations between serum YKL-40 level with demographic characteristics as well as biochemical measurements including serum creatinine, blood urea nitrogen, erythrocyte sedimentation rate, C-reactive protein and anti-cyclic citrullinated peptide.

**Conclusion:**

Our study revealed higher serum YKL-40 levels in RA patients compared to healthy controls, which correlated positively with disease activity. Therefore, YKL-40 can be considered as a novel biomarker for disease activity estimation in RA.

Rheumatoid arthritis (RA) is a chronic autoimmune inflammatory synovitis that affects approximately 1% of the population and contributes to functional disability (1). The etiology of RA is unknown; however, autoimmune mechanisms play a key role in the pathogenesis of the disease (2). It is indicated that chronic inflammation in RA leads to synovial proliferation associated with cartilage and bone resorption (3). It has been mentioned that biochemical markers of joint tissue metabolism and disease activity would be very useful for monitoring the disease course in RA patients (4). Several biochemical markers of joint metabolism have been studied in patients with RA. Up to now, the erythrocyte sedimentation rate (ESR) and the serum C reactive protein (CRP) have been the most widely used serological parameters for long term monitoring of disease activity and severity of RA (5, 6). 

However, there are differences between clinical inflammation and the level of ESR and serum CRP, and these parameters can be normal in patients with apparently active joint inflammation (5, 6). Therefore, new markers predicting clinical course and disease prognosis as well as following the response to treatments are of importance.

YKL-40 is a 40-kDa heparin-human cartilage glycoprotein-39 without enzymatic activity that is secreted by various cell types in the arthritic joint (5). Its name derives from the one letter code for the three terminal amino acids - tyrosine (Y), lysine (K) and leucine (L) (7). It is a major protein secreted by chondrocytes in vitro and in vivo YKL-40 identified in the chondrocytes from arthritic knee joints (8-14). Furthermore, YKL-40 levels in serum and synovial fluids increased in joint diseases like RA (7, 15, 16) and osteoarthritis (OA) (17, 18), indicating that YKL-40 is a marker of inflammation and tissue remodeling or degradation (6); whereas YKL-40 is not observed in normal joints. Previous studies showed that serum YKL-40 levels correlated with clinical, laboratory, and ultrasonography parameters of disease activity in RA patients (5, 6, 15, 16). The aim of the present study was to examine serum YKL-40 levels in patients with RA compared to healthy subjects and to search a correlation between serum YKL-40 concentrations with disease characteristics in RA.

## Methods

In current study, 60 patients aged 30 to 80 years with the diagnosis of RA, with disease duration ≥ 6 months, according to the ACR/EULAR 2010 criteria (19, 20) were selected consecutively from the outpatient rheumatology clinic of Tabriz University of Medical Sciences between September 2017 and February 2018. Also, 30 age- and sex matched healthy controls with no inflammatory rheumatic disease were included. The exclusion criteria were as follows: renal disease, liver disease, thyroid and/or parathyroid disorders, cardiovascular disease, diabetes mellitus, Cushing syndrome, any other autoimmune diseases or overlap syndromes, malignancies, pregnancy and lactation, cigarette smoking or alcoholism, taking oral contraceptive pills, and using vitamins, antioxidants, or other dietary supplements during the past 2 months prior to enrollment. This study was approved by the Ethics Committee of Tabriz University of Medical Sciences (Iran) and written informed consent was obtained from all subjects before inclusion in the study. At baseline, all participants were examined by a rheumatologist and the disease activity score 28 (DAS-28) measure of disease activity was recorded for patients with RA (21). Ranges of the DAS-28 score correspond with disease activity. A DAS-28 score of < 2.6 indicates remission, score of 2.6 to 3.2 indicates low disease activity, score of 3.2 to 5.1 indicates moderate disease activity and score of above 5.1 is considered high disease activity. 

Five mL of venous blood samples was obtained after 12-hour overnight fasting. The serum samples were separated from whole blood and were kept at −70°C until biochemical analysis. Serum YKL-40 (MyBioSource Inc., San Diego, CA, USA), anti-cyclic citrullinated peptide (anti-CCP) (Medizym., Berlin, Germany), and C-reactive protein (CRP) (Monobined Inc., Lake Forest, CA) levels were measured by ELISA according to the manufacturer’s recommendations, using an ELISA plate reader (Model stat fax 2100, Awareness, Ramsey, MN). Serum creatinine, blood urea nitrogen (BUN), and rheumatoid factor (RF) were measured by the standard enzymatic colorimetric method (Pars Azmoon Co, Tehran, Iran) with an automated chemical analyzer (Abbott analyzer, Abbott laboratories, Abbott Park, North Chicago, IL). The erythrocyte sedimentation rate (ESR) was measured using whole blood and complete blood counts with differential counts analyzed by the H1-Technicon blood cell counter. 

Statistical analysis was performed using SPSS software version 18.0 (SPSS, Inc., USA). Normality of variables distribution was evaluated using the Kolmogorov-Smirnov test. Variables not normally distributed were analyzed using nonparametric tests. Categorical and normally distributed quantitative variables were displayed as numbers (percentages) and means ± SD, respectively. Non-normally distributed quantitative variables were presented as median (interquartile range). Between group comparisons were made by χ^2^, independent-sample t test, and one-way analysis of variance (ANOVA), as appropriate. Correlations between variables were analyzed by Pearson correlation test or Spearman rank correlation analysis. A receiver operating characteristic (ROC) curve analysis was performed to identify the optimal cut-off points of serum YKL-40 levels for predicting RA. 

The area under the curve (AUC) value was calculated to determine the accuracy of the test. A p<0.05 was considered statistically significant.

## Results

The general, clinical and biochemical characteristics of study subjects are shown in table 1. The mean±SD age and duration of disease in patients were 54.86±11.65 and 1.99±1.22 years, respectively. The mean±SD disease activity and RF in patients were 4.16±1.77 and 49.63±28.07 IU/ml, respectively. The severity of disease according to the DAS-28 was remission in 11 (18.4%) patients, mild in 8 (13.3%), moderate in 21 (35%), and high in 20 (33.3%) patients. No significant differences were observed in general characteristics between RA patients and control subjects. 

**Table 1:** Characteristics of RA patients (n=60) and control subjects (n=30)

**Characteristics**	**RA patients**	**Control group**	**P** *
Sex, n (%)			
Male Female	5 (8.3) 55 (91.7)	5 (16.7) 25 (83.3)	0.236
Age (year)	54.86±11.65	50.71±3.72	0.07
Creatinine (mg/dL)	1.00±0.21	0.99±0.19	0.331
BUN (mg/dL)	32.33±10.79	27.96±12.12	0.093
ESR (mm/hr)	38.55±23.03	-	-
CRP (mg/L)	14.93±8.64	-	-
Anti-CCP (U)	398.5 (200, 1280)	-	-
RF (n [%])			
+1 +2 +3 +4	27 (45) 20 (33.3) 11 (18.3) 2 (3.3)	- - - -	-
DAS-28	4.16±1.77	-	-

RA: rheumatoid arthritis, BUN: blood urea nitrogen, ESR: erythrocyte sedimentation rate, CRP: C-reactive protein, Anti-CCP: anti-cyclic citrullinated peptide antibody, RF: rheumatoid factor, DAS-28: disease activity score-28.

Data were presented as mean±SD and median (25^th^ and 75^th^ percentiles) for normally and non-normally distributed quantitative measures, respectively or number (percent) for categorical variables.

* p values indicate comparison between groups (χ2 or independent-sample t test, as appropriate).

**Figure 1:**
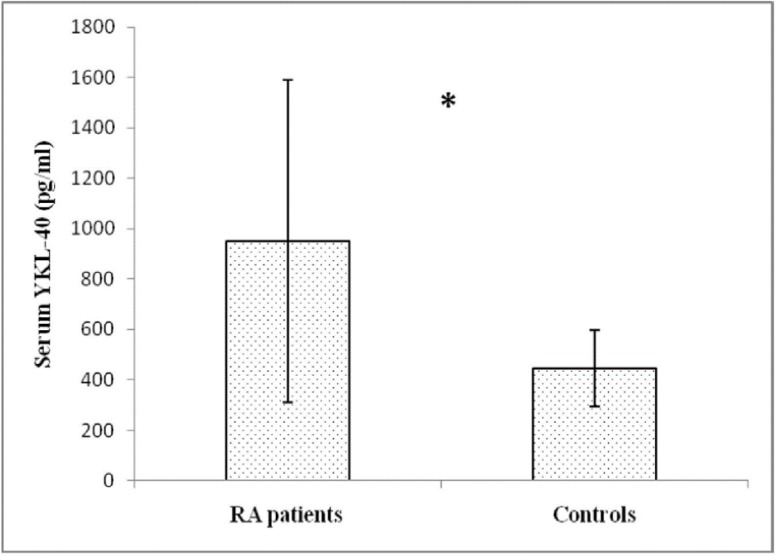
Serum concentrations of YKL-40 in RA patients (n=60) and control group (n=30). Values are means±SD (*Independent-sample t test for between group comparison, P<0.001)

As presented in figure 1, serum YKL-40 level was significantly higher in RA patients (951.63±639.98 pg/mL) compared to healthy controls (444.92±150.37 pg/mL) (p<0.001). 

**Figure 2:**
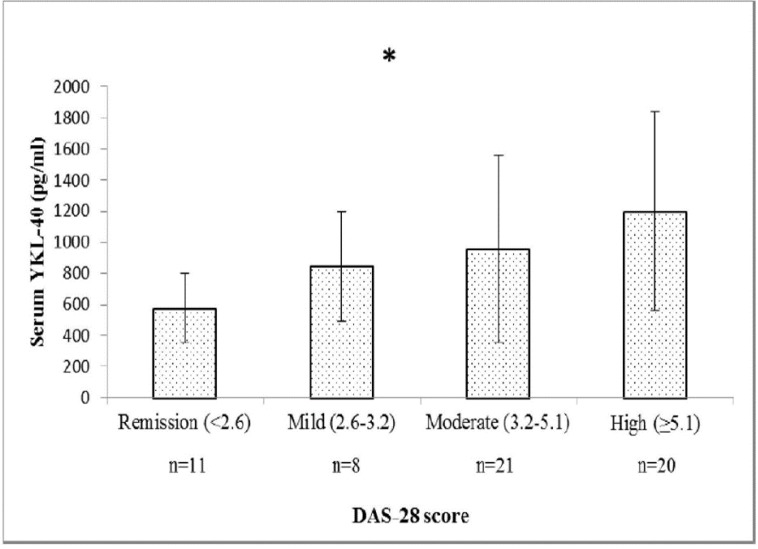
Comparison of serum YKL-40 levels based on DAS-28 score in RA patients (n=60). Values are means ± SD (*One-way ANOVA for between group comparison, P=0.066)

Figure 2 demonstrates serum YKL-40 levels in RA patients based on DAS-28 score. There was no significant difference in serum YKL-40 level according to the activity of disease (p>0.05).

**Figure 3:**
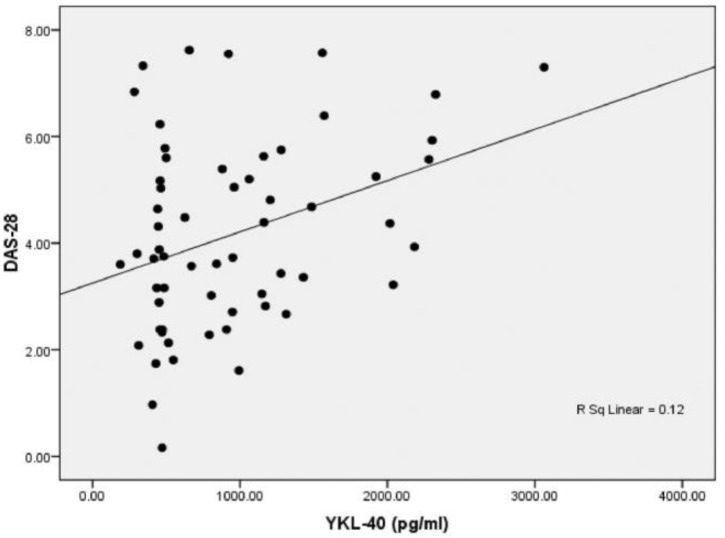
Correlation between serum concentrations of YKL-40 and DAS-28 in RA patients (n=60)

**Figure 4:**
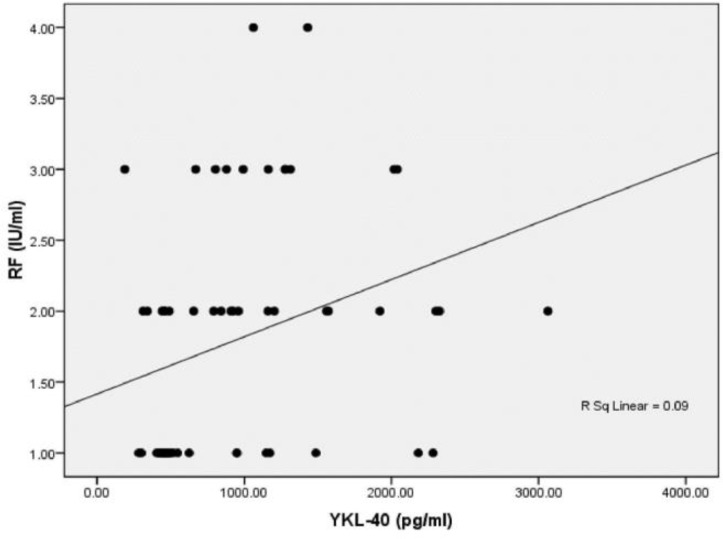
Correlation between serum concentrations of YKL-40 and RF in RA patients (n=60)

As illustrated in figures 3 and 4, there were significant positive correlations between serum YKL-40 level with disease activity (r=0.347, p=0.007) and RF (r=0.396, p=0.002). 

There were no significant correlations between serum YKL-40 level with demographic characteristics as well as biochemical measurements including serum creatinine, BUN, ESR, CRP and anti-CCP (p>0.05). 

**Figure 5:**
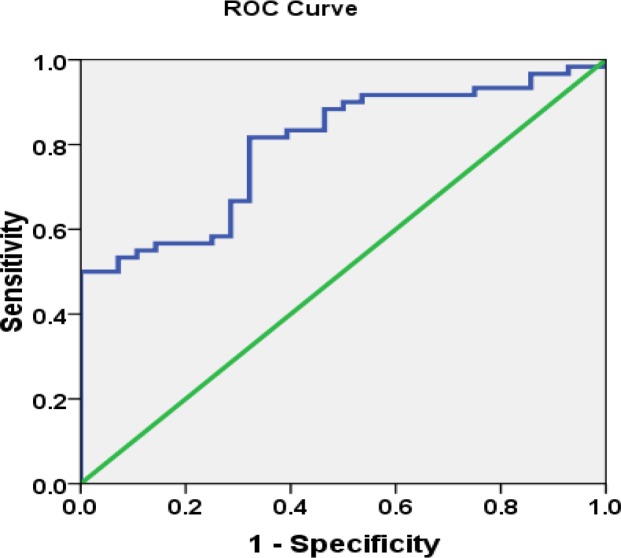
Receiver operating characteristic (ROC) curve analysis for predictive values of serum concentrations of YKL-40 in RA. The area under the ROC curve is 0.797; 95% CI: 0.704–0.890, p<0.001

The ability of serum YKL-40 concentrations to distinguish patients with RA from those without RA was assessed using ROC curve analysis (figure 5). ROC curve was well above the diagonal, indicating good sensitivity and specificity. 

The ROC curve for the presence of RA diagnosis had an AUC of 0.797 (95% CI: 0.704–0.890, p<0.001) which indicated a high probability of correctly prediction of RA.

## Discussion

RA is an autoimmune disorder in which chronic active inflammation leads to synovial proliferation of cartilage and bone (22). A wide range of serum markers were used to measure disease activity in RA patients, however, there was no single biomarker that could demonstrate the biology of disease and to help predict clinical course. Therefore, there is still a need for specific novel biomarker of synovial inflammation and disease activity. YKL-40 is considered as a possible new biomarker. It is assumed that YKL-40 is a candidate autoantigen in RA, suggesting to play a pathogenic role in the inflammatory process and joint destruction.

In the present study, we found that serum YKL-40 was elevated in patients with RA compared to healthy subjects and correlated with the disease activity as measured by DAS-28 score which was similar to previous studies (5, 6, 12, 15, 23, 24). In another study, it was shown that patients with clinically active RA that became inactive during the one-year study period had a significant decrease in serum YKL-40 level, whereas patients whose RA remained clinically active showed an increase in serum YKL-40 level. This study confirmed that serum YKL-40 was influenced by the disease activity in RA and behaved as another acute phase reactant, like serum hsCRP and ESR (16). These findings were also supported by previous report in which YKL-40 levels were high in severe RA (25). Furthermore, YKL-40 can have a role in cartilage destruction in arthritic joints and that serum YKL-40 may reflect a combination of cartilage metabolism and a more local aspect of the inflammatory process than serum CRP and ESR in patients with RA. It has been noticed that different cells including synovial cells, chondrocytes, osteoblasts, macrophages, and neutrophils produce serum YKL-40 in RA patients, but it is difficult to recognize which cells are responsible for the increase in serum YKL-40 levels.

Based on our study, there were no significant correlations between serum YKL-40 with serum CRP and ESR which was in contrast with previous investigations (15, 16, 23, 25, 26). Several pathological conditions affect these parameters; therefore, these laboratory parameters are non-specific indicators of synovial inflammation (7). Our study was consistent with Johansen et al.’s (27) study who also did not find positive correlations between serum CRP and YKL-40 levels in OA patients. This discrepancy between our findings with previous studies might be due to the differences in studied population, disease duration, baseline disease activity and YKL-40 status as well as type, dosage and duration of medical therapies. The present study has the following limitations: (i) the relatively small sample size and (ii) patients were treated with immunosuppressive therapies, such as steroids, at the time point of investigation and blood sampling which may affect the results. The strength of this study was that we had an age- and sex-matched control group and compared mean serum YKL-40 levels between patients with RA and normal subjects. 

In conclusion, our study revealed higher serum YKL-40 levels in RA patients compared to healthy controls, which correlated positively with disease activity. Therefore, YKL-40 can be considered as a novel biomarker for disease activity estimation in RA. 
